# Draft Genome Sequences of Type Strains of *Adlercreutzia muris* and *Ellagibacter urolithinifaciens*, Belonging to the Family *Eggerthellaceae*

**DOI:** 10.1128/MRA.01306-19

**Published:** 2019-11-21

**Authors:** Nicolas Danylec, Dominic A. Stoll, Melanie Huch

**Affiliations:** a Max Rubner-Institut, Federal Research Institute of Nutrition and Food, Department of Safety and Quality of Fruit and Vegetables, Karlsruhe, Germany; Queens College

## Abstract

Here, we report the annotated draft genome sequences of two type strains belonging to the family *Eggerthellaceae* within the class *Coriobacteriia* (phylum *Actinobacteria*), *Adlercreutzia muris* WCA-131-CoC-2 (= DSM 29508 = KCTC 15543) and *Ellagibacter urolithinifaciens* CEBAS 4A (= CCUG 70284 = DSM 104140).

## ANNOUNCEMENT

Species of the family *Eggerthellaceae* are typical members of the mammalian gut and have been isolated from, e.g., humans (1, 2), mice (3–5), and sheep (6). Several *Eggerthellaceae* species have been reported to metabolize secondary plant compounds, especially polyphenols like ellagic acid (2), daidzein (3), and resveratrol (7). *Ellagibacter urolithinifaciens* CEBAS 4A^T^ was isolated from the feces of a 41-year-old healthy male during the search for a bacterial strain that is able to metabolize ellagic acid. This strain is capable of metabolizing ellagic acid into isourolithin A (2). The type strain of Adlercreutzia muris was isolated as Enterorhabdus muris from the colonic content of a wild-type C57BL/6 mouse (5, 8). The genus Enterorhabdus showed only a low genetic divergence with the genus *Adlercreutzia* and has to be seen as a later heterotypic synonym of the previously described genus *Adlercreutzia* (9, 10).

In this study, we announce the annotated draft genome sequences of the type strains of *Adlercreutzia muris* and *Ellagibacter urolithinifaciens.* With this announcement, we provide the remaining annotated draft genome sequences of type strains of the family *Eggerthellaceae* with standing names in nomenclature (11).

The following type strains were obtained from the German Collection of Microorganisms and Cell Cultures (DSMZ): *A. muris* DSM 29508 and *E. urolithinifaciens* DSM 104140. Strains were cultured, and genomic DNA was isolated as previously described (1, 12, 13). *Adlercreutzia muris* DSM 29508^T^ was cultured (37°C) under anaerobic conditions of N_2_-CO_2_ (80:20) in flushed brain heart infusion medium (Merck) supplemented with 0.5% yeast extract, 0.05% l-cysteine monohydrochloride (Roth), 1 mg ml^−1^ resazurin sodium salt, 2.5 mg liter^−1^ heme solution, and 2 μg ml^−1^ vitamin K_1_ solution (Sigma-Aldrich). *Ellagibacter urolithinifaciens* DSM 104140^T^ was cultured (37°C) under anaerobic conditions of N_2_-CO_2_ (80:20) in flushed anaerobe basal broth (Oxoid). DNA extraction was done using a Qiagen blood and tissue kit. DNA was quantified with a double-stranded DNA (dsDNA) high-sensitivity (HS) assay on a Qubit version 2.0 fluorometer (Thermo Fisher Scientific), according to the manufacturer’s instructions, and adjusted to a concentration of 0.2 ng μl^−1^.

The sequencing library was constructed using a Nextera XT DNA library prep kit and a Nextera XT index kit (Illumina). Whole-genome shotgun sequencing was performed on an Illumina MiSeq benchtop sequencer using a 500-cycle v2 kit (read length, 2 × 250 bp). In total, 1,469,853 reads were generated for *A. muris* DSM 29508^T^, while 1,067,866 reads were generated for *E. urolithinifaciens* DSM 104140^T^. Data processing was done as previously described (12, 13). Sequence reads were quality trimmed using Trimmomatic version 0.39 (14) and assembled using SPAdes version 3.13.1 with default settings in the “careful” mode (15, 16). The estimated insert sizes of *A. muris* DSM 29508^T^ and *E. urolithinifaciens* DSM 104140^T^ were 255.724 bp and 297.597 bp, respectively. Adequate trimming was verified by mapping the adapter sequences to the assembled contigs using Bowtie 2 version 2.3.3.1 (17). To eliminate sequence contamination, the contigs were aligned to the genome of coliphage phi-X174 (GenBank accession number NC_001422) using a BLASTn search (18). All contigs of <500 bp were manually excluded, and renaming of contigs was done using awk (19). To calculate genome coverage of each strain, trimmed reads were mapped against remaining contigs by Bowtie 2. In the case of *A. muris* DSM 29508^T^, 99.15% of 1,463,425 trimmed reads could be mapped against the respective contigs, whereas in the case of *E. urolithinifaciens* DSM 104140^T^, 99.18% of 1,064,956 trimmed reads could be mapped against the contigs. Draft genome sequences were annotated using the automated NCBI Prokaryotic Genome Annotation Pipeline (20). The assembly metrics and annotated features of both type strains are given in Table 1. ResFinder version 3.2 (21) was used to evaluate the draft genome sequences for antimicrobial resistance (AMR) genes. For both strains, no AMR gene was identified.

**TABLE 1:** Accession numbers, assembly metrics, and annotated features of the type strains of *Adlercreutzia muris* and *Ellagibacter urolithinifaciens*

Bacterial species (strain)	Genome size (bp)	No. of contigs (avg coverage [×])	*N*_50_ (bp)	Total no. of genes	G+C content (%)	GenBank accession no.
*Adlercreutzia muris* (DSM 29508)	2,765,182	94 (134.19)	79,375	2,303	65.1	WAJS00000000
*Ellagibacter urolithinifaciens* (DSM 104140)	2,462,117	81 (127.67)	100,872	2,161	60.4	WAJR00000000

### Data availability.

This whole-genome shotgun project, including raw reads of *A*. *muris* DSM 29508^T^ and *E*. *urolithinifaciens* DSM 104140^T^, has been deposited at DDBJ/ENA/GenBank under BioProject number PRJNA574580. The versions described in this publication are the first versions and are listed in Table 1. The raw reads of *A*. *muris* DSM 29508^T^ have been deposited under SRA accession number SRX6974936, while the raw reads of *E*. *urolithinifaciens* DSM 104140^T^ have been deposited under accession number SRX6974937.
